# A Multi-Layered 3D NDT Scan-Matching Method for Robust Localization in Logistics Warehouse Environments

**DOI:** 10.3390/s23052671

**Published:** 2023-02-28

**Authors:** Taeho Kim, Haneul Jeon, Donghun Lee

**Affiliations:** Mechanical Engineering Department, Soongsil University, Seoul 06978, Republic of Korea

**Keywords:** 3D NDT scan-matching, localization, Isaac sim, indoor navigation, 3D point-cloud map

## Abstract

This paper proposed a multi-layered 3D NDT (normal distribution transform) scan-matching approach for robust localization even in the highly dynamic environment of warehouse logistics. Our approach partitioned a given 3D point-cloud map and the scan measurements into several layers regarding the degree of environmental changes in the height direction and computed the covariance estimates for each layer using 3D NDT scan-matching. Because the covariance determinant is the estimate’s uncertainty, we can determine which layers are better to use in the localization in the warehouse. If the layer gets close to the warehouse’s floor, the degree of environmental changes, such as the cluttered warehouse layout and position of boxes, would be significantly large, while it has many good features for scan-matching. If the observation at a specific layer is not explained well enough, then the layer for localization can be switched to other layers with lower uncertainties. Thus, the main novelty of this approach is that localization robustness can be improved even in very cluttered and dynamic environments. This study also provides the simulation-based validation using Nvidia’s Omniverse Isaac sim and detailed mathematical descriptions for the proposed method. Moreover, the evaluated results of this study can be a good starting point for further mitigating the effects of occlusion in warehouse navigation of mobile robots.

## 1. Introduction

Accurate and robust localization is essential for navigating autonomous mobile robots. The scan-matching methods play a significant role in the localization as well as simultaneous localization and mapping (SLAM). However, most existing localization approaches assume that the environment is static, which does not hold for most practical application domains such as warehouse logistics. Thus, in this paper, we introduced a multi-layered 3D NDT-based scan-matching algorithm to improve the robustness of the scan-matching task, which is highly related to localization accuracy, in very sophisticated indoor environments. The term robustness refers to the ability of the scan-matching method to perform accurately and consistently under different environmental conditions, such as changes in product or layout and other disturbances. In this study, 3D LiDAR was preferred for 3D NDT scan-matching. However, there have been many studies regarding localization using other sensors and robotic platforms. Regarding localization in the logistics warehouse, Ekici et al. [1] proposed a drone-based approach for indoor positioning and product counting using virtual fiducial markers in warehouse environments. However, this method relies on the availability and placement of these markers, which may not be practical or feasible in all warehouse environments. Moreover, drones may not be suitable for all warehouse applications, especially those involving large and heavy items. Zeng et al. [2] proposed a LiDAR positioning algorithm based on ICP and artificial landmarks assistance. While this approach may be effective in some environments, it is limited by the need for pre-placed artificial landmarks and may not be able to adapt to highly dynamic and cluttered environments. Additionally, the algorithm’s performance may be affected by the quality and density of the LiDAR data, which may vary depending on the device and environment. Noh et al. [3] discussed the limitations of vision sensors, such as their sensitivity to lighting changes and the complexity of processing the large amounts of data generated by high-resolution cameras. Thus, the study focused on developing an efficient 3D Lidar-based SLAM method for mobile robots in indoor environments. Moreover, the use of 3D LiDAR was preferred in our study over 2D LiDAR due to its capability to provide a more comprehensive and accurate representation of the environment. As stated by Liu et al. [4], 3D LiDAR can generate 360-degree, dense, and high-resolution point clouds, making it more suitable for precise localization and mapping in complex and dynamic environments. Moreover, Basavanna et al. [5] demonstrated that the fusion of 3D LiDAR and RGB-D cameras could enhance the quality of mapping, which is essential for applications such as autonomous driving. Although the mobile robot in this study moved on flat ground, 3D LiDAR was preferred due to its ability to provide richer and more detailed data, allowing for more robust and accurate perception and decision-making. There are two most popular algorithms for the scan-matching of iterative closest point (ICP) and NDT, which [6,7,8,9] are employed to minimize the difference between two-point clouds. In additions, Arun et al. [6] proposed a method for fitting two 3D point sets based on least-squares, which can be used for object recognition and registration. Besl et al. [7] introduced a method for registering 3D shapes based on ICP. Chen et al. [8] presented a method for object modeling by registering multiple range images, which can be used for 3D shape reconstruction and recognition. Zhang et al. [9] proposed an iterative point-matching method for registering free-form curves and surfaces, which can be used for 3D shape alignment and reconstruction. As the size of the 3D point-cloud map increases, NDT is used more in consideration for real-time localization in terms of computational efficiency because a set of normal distributions represents the map [10], and there is no need for the most time-consuming nearest neighbor search performed by comparing each point to find the point-to-point correspondences. Thus, NDT can better cope with slight environmental changes [10,11,12] than the ICP, while the NDT sometimes falls into a local minimum due to the low accuracy of the initial pose [13]. Biber and Straßer [10] introduced the normal distributions transform (NDT) method for scan-matching, which is capable of dealing with scan data of complex environments. In the study by Magnusson et al. [11], the scan registration for autonomous mining vehicles using 3D-NDT was conducted in an underground mine environment where the scans were affected by dust, vibrations, and dynamic obstacles. The study confirmed that the 3D-NDT scan-matching method was robust enough to handle these environmental changes and provide accurate registration. Akai et al. [12] considered the effect of weather and lighting conditions on the performance of the 3D-NDT scan-matching method. They proposed combining 3D-NDT with road marker matching to improve localization accuracy under such environmental changes.

It is also reported that the error of NDT when the scan-matching fails is more significant than that of ICP scan-matching [8]. In addition, the convergence performance of NDT depends on the appearance of the environment, and the localization accuracy decreases in the case where there are differences between the environment and map [14,15] since the 3D NDT scan-matching algorithm assumes that the environment between scans is static, meaning that the objects and structure of the environment do not change. However, in real-world scenarios, this is often different. For example, in robotics applications, objects may move or change their position between scans or add or remove objects from the environment in a mapping scenario. These changes can cause significant errors in the final results of the 3D NDT scan-matching, as the algorithm is not designed to handle these highly changeable environments. Moreover, a higher occlusion rate [16] would generally result in a higher NDT scan-matching error. This is because the algorithm relies on finding correspondences between points in the scans, and in cluttered environments, it can be challenging to determine which points belong to the same object or structure. Additionally, changes in the map between scans can cause mismatches and further reduce the accuracy of the 3D NDT scan-matching [17,18]. In these scenarios, alternative algorithms or methods may be required to improve the accuracy of the scan-matching.

Several advancements have been made to improve the accuracy and efficiency of the original NDT method. One study [10] presented an improved 3D NDT for 3D scan-matching by modifying the 2D NDT and accelerating the process using dual resolution with two different cell sizes. One such improvement involves dividing the point cloud into multiple sections and using a 2D NDT algorithm in marker-free registration for 3D laser scanning [19]. This study proposed three modifications to the original NDT, including a rough-to-fine strategy, multiple slices, and the Levenberg–Marquardt algorithm for optimization.

Recently, Cihan [20] introduced a new multi-layer normal distribution transform (ML-NDT) concept for 3D scan-matching. The ML-NDT partitions the entire point cloud map, similar to the concept of an octree representation. The octree representation is a hierarchical data structure that divides a 3D space into smaller and smaller parts recursively until a specific criterion is met. Similarly, the ML-NDT partitions the point cloud into multiple layers using a 2D NDT algorithm, allowing for more efficient and accurate scan-matching. This way, the ML-NDT reduces the complexity of the 3D scan-matching problem and speeds up the process. The results from simulations and experiments support the claim that the ML-NDT method is suitable for fast and long-range scan-matching applications. However, what about the environmental changes occurring within a specific height area, such as a warehouse? The ML-NDT approach can handle environmental changes more efficiently, as each layer can be treated as a separate 2D map. However, it may be less effective if the changes are mainly confined to a specific height range, as the ML-NDT partitions the entire point cloud into multiple layers based on a 2D NDT algorithm, which may not accurately capture the height-based changes.

Thus, in this work, we partitioned a given 3D point-cloud map and the 3D lidar scan measurements into several layers to perform the 3D NDT scan-matching by separating the static and dynamic zones, since slicing the 3D point-cloud map in the height direction to separate significant environmental changes can be effective if the environmental changes are mainly confined to a specific height range near the ground as in the warehouse. Furthermore, this approach can provide a clear separation between the affected and unaffected areas, making detecting and processing changes easier. That is, the proposed method separates the point cloud based on changes in height, while in the ML-NDT approach, the layers are generated using a 2D NDT algorithm, which may result in a different partitioning of the point cloud. After the slicing, we computed the covariance estimates of 3D NDT scan-matching for each layer because the covariance determinant is the estimate’s uncertainty of the scan-matching result. Finally, we determined which layers are better for localizing the logistics warehouse’s mobile robot (MR). If the layer is close to the warehouse’s bottom floor, the degree of environmental dynamics, such as the cluttered warehouse layout and pose of warehouse products, would be significantly large, while it has many good features for scan-matching. If the observation at a specific layer is not explained well enough, then the layer for localization can be switched to other layers with lower uncertainties. Thus, this paper makes the following research contributions.

1. Complete mathematical description for the multi-layered 3D NDT scan-matching is provided;
2. Robustness of the 3D NDT scan-matching-based localization can be greatly improved by identifying the layers that significantly cause the uncertainties in scan-matching using the entire map;
3. Accurate localization in the warehouse’s cluttered and dynamic logistics environments can be achieved;
4. Robustness and accuracy of a proposed method are thoroughly evaluated by comparing standard 3D NDT scan-matching through the domain randomization technique of Nvidia Isaac Sim, which powers a photorealistic and physically accurate virtual environment.

The rest of this paper is composed as follows. Section 2 describes the detailed method of the proposed multi-layered 3D NDT scan-matching algorithm. In Section 3, we present the simulation and performance evaluation. Then, in Section 4, we conclude the paper.

## 2. Methods

This section describes a single lidar scan’s multi-layered 3D Normal Distributions Transform (NDT). This is meant to be the central contribution of the paper. In our work, we assume that a static map with multiple map layers is given and a lidar scan has the same number of scan layers as map layers. Additionally, it is assumed that each map layer and its corresponding scan layer have the same height range. Then, the multi layered 3D NDT scan-matching evaluates the distributions of all scan layers subdivided from a single lidar scan by summing the local normal distribution in all cells in each map-layer. 

The given multi-layered 3D point-cloud map, represented as a voxel map, was built beforehand from a 3D point-cloud after a voxel grid (VG) filtering. For an *i*th map-layer, collect all points $p_{j,k=1\ldots N}$ contained in the *j*th cell. And then calculate the mean $p_{i,j}^{m}$ of each voxel from Equation (1).

(1) $$p_{i,j}^{m}=1/N\sum_{k}^{N}p_{i,j,k}$$

After calculating the covariance matrix of each cell from Equation (2), the probability of a single point in the *j*th cell can be modeled by the normal distribution of $N_{i,j}\left(p_{i,j}^{m},\sum_{i,j}\right)$. To start scan-matching, *l*th point ${p^{\prime }}_{i,j,l}$ from an *i*th scan-layer assigned to *j*th cell is assigned to a PDF as shown in Equation (3) depending on which cell the point is in. Finally, the matching score of *i*th layer, a total sum of the scan-matching’s probability for *i*th scan-layer with *i*th map-layer, can be calculated from Equation (4).

(2) $$\sum_{i,j}=1/N\sum_{k}^{N}\left(p_{i,j,k}-p_{i,j}^{m}\right){\left(p_{i,j,k}-p_{i,j}^{m}\right)}^{T}$$

(3) $$p\left({p^{\prime }}_{i,j,l}\right)∼\exp\left\{-1/2{\left({p^{\prime }}_{i,j,l}-p_{i,j}^{m}\right)}^{T}\sum_{i,j}^{-1}\left({p^{\prime }}_{i,j,l}-p_{i,j}^{m}\right)\right\}$$

(4) $$p_{i}\left({p^{\prime }}_{i}\right)=\sum_{j}^{}\sum_{l}^{}\exp\left\{-1/2{\left({p^{\prime }}_{i,j,l}-p_{i,j}^{m}\right)}^{T}\sum_{i,j}^{-1}\left({p^{\prime }}_{i,j,l}-p_{i,j}^{m}\right)\right\}$$

To select scan-matching layers to be rejected in localization, let us first consider a finite non-empty set $P=\left\{p_{1},\;\;\ldots \;\;,p_{M}\right\}\;$ of scan-matching scores for all layers obtained from Equation (4). According to surjection f:I→P, I is an indexing set for P. A parameter for the scan-matching threshold, κ∈ℝ, is also considered here. Then, the Equation (5) can be considered to reject scan-matching layers, adversely affecting localization accuracy. Here, it should be noted that the threshold κ, which should be determined through experiments, will be discussed in Section 3.

(5) $$P^{\prime }={\left\{\forall p_{i}\left[\inf P\;\vee \forall p_{i}\left[p_{i}\leq \kappa \right]\right]\right\}}^{C},\;\;∴f:I^{\prime }\to P^{\prime }$$

**Figure 1:**
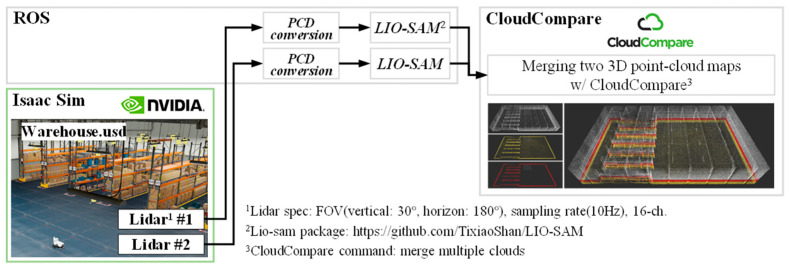
Framework for 3D point-cloud map slicing after SLAM process using LIO-SAM [21,22] with dual scan measurements from two 3D lidar sensors.

**Figure 2:**
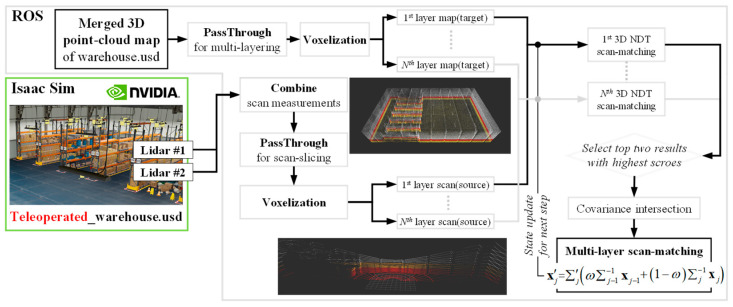
Framework of a multi-layered 3D NDT scan-matching based on a 3D point-cloud map and scan measurement sliced in the height direction.

Now we have $I^{\prime }$, a reordered indexing set, after rejecting all unsatisfactory scan-matching layers. For layers in $I^{\prime }$, let us consider now we have the two most confident scan-matching estimates, and then apply the weighted sum between the two scan-matching results as shown in Equation (6).

(6) $$x=x_{i}+{\sum^{\prime }}_{i}{\left({\sum^{\prime }}_{i}+{\sum^{\prime }}_{i+1}^{}\right)}^{-1}\left(x_{i+1}-x_{i}\right)$$

where, ${\sum^{\prime }}_{i}\in {\mathbb{R}}^{3\times 3}$ and $x_{i}\in {\mathbb{R}}^{3}$ denotes the state covariance matrix and the estimated state vector at *i*th layer’s scan-matching and then $x\in {\mathbb{R}}^{3}$ denotes the resultant state. In other words, when the lidar scan comes in, it goes through this process every time. For infinitely large covariance from the *i* + 1th layer, the estimated state of *i* + 1th layer will be rejected from Equation (6) and vice versa. Moreover, the *i*th state will be returned as a resultant state of the multi-layered 3D NDT scan-matching. In addition, in the KF, the posterior state and covariance affect state prediction in the next step, but the NDT scan-matching already uses the estimated state from the previous step as the initial state. Thus, in the fusion of the two scan-matching results as in Equation (6), there is no need to consider the estimation uncertainty in the previous step additionally. In the next section, based on Figure 1 and Figure 2, the simulation framework of a multi-layered 3D NDT scan-matching method is described.

## 3. Simulation and Performance Evaluation

### 3.1. Simulation Framework of a Multi-Layered 3D NDT Scan-Matching Method

Figure 1 and Figure 2 show two frameworks for the ML-3D NDT scan-matching process proposed in this study. In the case of a warehouse, it is not easy to map a 3D point-cloud map of a high area of a warehouse with a single 3D lidar. Therefore, Figure 1 explains the framework that performs SLAM and mapping of LIO-SAM with dual 3D lidar and then merges them with CloudCompare software. In addition, Figure 2 explains the ML-3D NDT scan-matching process after slicing the entire 3D point-cloud map and 3D scan measurements obtained in this way into n layers through the PassThrough filter.

Regarding the voxelization process performed before the layer-by-layer 3D NDT scan-matching, a map is divided into small 3D voxels, and a multivariate normal distribution with a mean and covariance matrix represents each voxel. In this study, a technique called covariance intersection [23,24,25], as described in Algorithm 1 (https://github.com/KIT-ISAS/data-fusion/blob/master/algorithms/covariance_intersection.py (accessed 20 January 2023) [26]), was used to obtain the representative covariance matrix of scan-matching at each layer by combining the covariance matrices of all distributions in a way that preserves their relative uncertainty. The result is a mean vector and covariance matrix representing the estimated state and overall uncertainty of the 3D NDT scan-matching at each layer.
**Algorithm 1** Pseudocode for the covariance intersection of multiple covariance matrices**Inputs:** layered 3D point-cloud scan data leaf size for voxelization **Outputs:** ${x^{\prime }}_{i}$: mean vector of *i*th layer ${\sum^{\prime }}_{i}$: covariance matrix of *i*th layer **Initialization process:** 1:    Create an instance of *CovarianceIntersection class* as *fuser*; 2:    Do the voxelization of layered 3D point-cloud scan data; 3:    Road sets of mean and covariance arrays from voxelization; 4:    Initialize *mean*(1) and *cov*(1) with mean and covariance of the 1st cell. **Covariance intersection process:** 1: for all cells (*j* = 2 …. *num_cell*) do; 2:     *current_mean* = *mean*(*j*), *current_cov* = *cov*(*j*); 3:    *fused_mean* = *mean*(*j* − 1), *fused_cov* = *cov*(*j* − 1); *4:    mean*, *cov* = *fuser.fuse*(*fused_mean, fused_cov, current_mean, current_cov*); 5: end for; 6: return a final *mean* and *cov* as the mean and covariance of *i*th layer, ${x^{\prime }}_{i}$ and ${\sum^{\prime }}_{i}$, respectively.

Equations (7) and (8) represent formulas to calculate the fused mean and covariance with the neighboring two cells. First, after initializing the mean and covariance with the first cell’s mean and covariance, fused mean and covariance can be obtained through Equations (7) and (8). Then, the fused mean and covariance are fused with the next cell’s mean and covariance recursively until index *j* reaches the number of cells.

(7) $${\sum^{\prime }}_{j}={\left(\omega \sum_{j-1}^{-1}+\left(1-\omega \right)\sum_{j}^{-1}\right)}^{-1}$$

(8) $${x^{\prime }}_{j}={\sum^{\prime }}_{j}\left(\omega \sum_{j-1}^{-1}x_{j-1}+\left(1-\omega \right)\sum_{j}^{-1}x_{j}\right)$$

where, ${\sum^{\prime }}_{j}\in {\mathbb{R}}^{3\times 3}$ and ${x^{\prime }}_{j}\in {\mathbb{R}}^{3}$ denotes the fused covariance and mean obtained in each covariance intersection loop. $\sum_{j}$ and $x_{j}$ denotes the covariance and mean of the *j*th cell in *i*th layer. And ω denote the optimization coefficient [26]. The prime notation of the superscript on the right means the fused result in each loop. Thus, ${\sum^{\prime }}_{j}$ and ${x^{\prime }}_{j}$ becomes ${\sum^{\prime }}_{i}$ and $x_{i}$ in the end of this fuse process.

### 3.2. Simulation Environment: Logistics Warehouse

A typical logistics warehouse is composed of several types of assets, including:

1. Racks and Shelving: The primary storage structure in a warehouse is to store boxes, pallets, and other items;
2. Conveyors: Used to move items and packages around the warehouse, including receiving docks, sorting lines, and shipping docks;
3. Forklifts: Used to move heavy items and pallets within the warehouse, including stacking and retrieving items from the racks;
4. Loading Bays: Areas designated for loading and unloading trucks, trailers, and containers;
5. Pick and Pack Stations: Areas designated for picking and packing items for shipment, typically equipped with conveyors, packing tables, and materials handling equipment.

**Figure 3:**
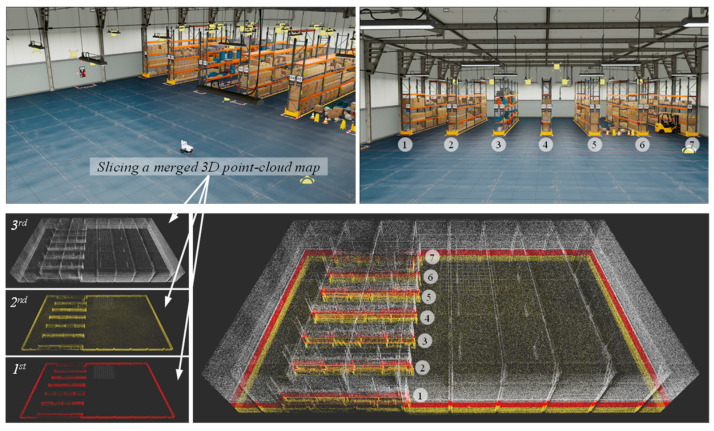
(**Top**) virtual warehouse environment, including two assets of full_warehouse.usd and Carter_v2.usd with proper lighting control in RTX real-time rendering mode, (**Bottom**) example of multi-layer slicing of a merged 3D point-cloud map obtained from LIO-SAM and dual 3D lidar.

**Figure 4:**
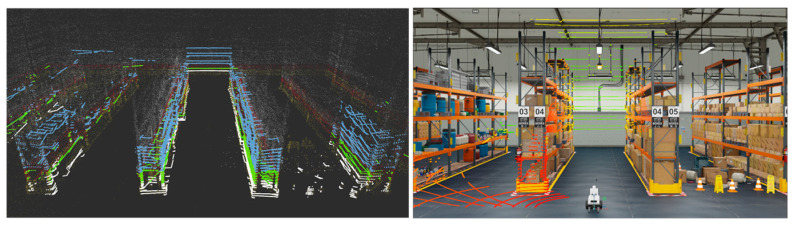
(**Left**) multi-layered scan measurement overlapped on the 3D point-cloud map of the full warehouse in ROS 3D visualization tool of RViz, (**right**) multi-layered 3D NDT scan-matching scene in Isaac Sim.

This study uses the Nvidia Omniverse Isaac sim to build a photorealistic virtual warehouse environment. We can use USD (Universal Scene Description) assets in Omniverse Isaac Sim to build a logistics warehouse. To create shelves for empty shelves, we can use USD geometry primitives such as boxes, cylinders, or planes and arrange them to form the shelves. To create racks for shelves filled with boxes, we can use USD models of boxes or other objects to represent the items stored on the shelves. We can also use USD light and camera primitives to create a believable environment and control the lighting and camera settings. In the following Figure 3 and Figure 4, the warehouse composed of the ‘full_warehouse.usd’ and ‘Carter_v2.usd’ is represented with the sliced 3D point-cloud maps for the multi-layered 3D NDT scan-matching-based localization. 

### 3.3. Comparative Study: Convergence Performance and Scan-Matching Robustness

**Figure 5:**
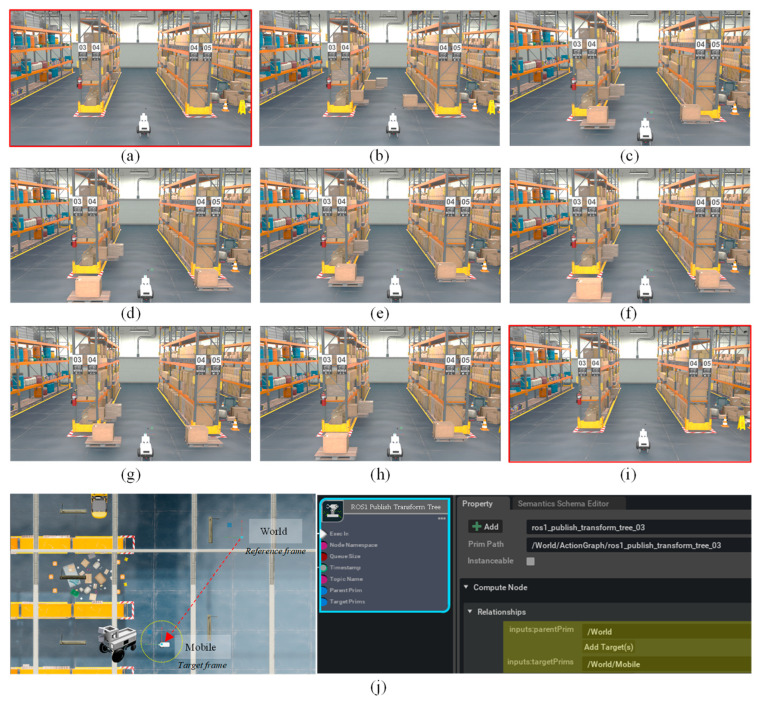
Multi-layered 3D NDT scan-matching scenario running in the Isaac Sim: (**a**) start w/o object teleport (same with original map); (**b**) start w/object teleport; (**c**–**h**) teleport events occurring every 10 s; (**i**) final scene identical with the original map; and (**j**) ground truth published from the Isaac Sim.

In this section, the following two simulation scenarios were devised to quantitatively compare and evaluate the robustness of the ML-3D NDT scan-matching method against significant environmental changes compared to the general 3D NDT scan-matching method and analyze the results. In both scenarios 1 and 2, object-teleport was set to occur in the first layer area. Figure 5 represents the multi-layered 3D NDT scan-matching scenario running in the Isaac Sim.

1. Scenario 1: Start 3D scan-matching-based localization without object-teleport -> When scan-matching starts, start the same as the original map, and then use Isaac Sim’s teleport to appear boxes on the pallet around MR and change its location at regular time intervals;
2. Scenario 2: 3D scan-matching-based localization starts with object-teleport -> The difference from scenario one is that scan-matching starts in a scene different from the original 3D point-cloud map through the teleport.

**Figure 6:**
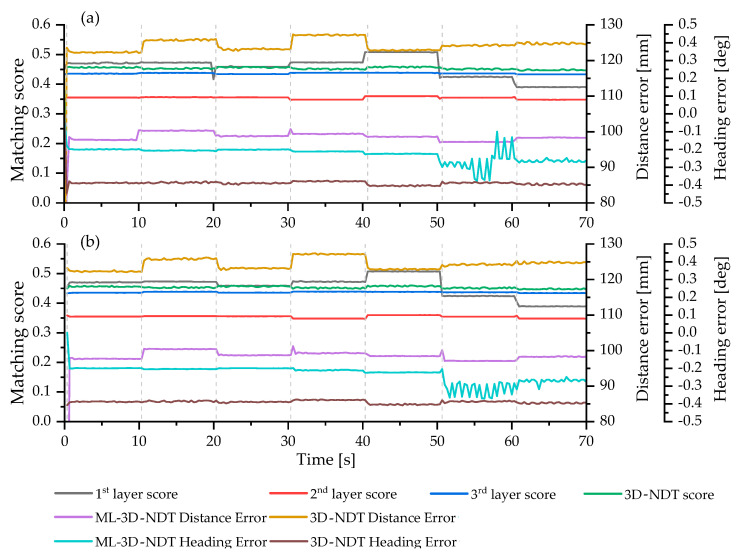
Result of the comparative study in terms of localization accuracy and layer-by-layer scan-matching score: (**a**) scenario 1-1; and (**b**) scenario 2-1.

In general, since the initial state accuracy of the NDT scan-matching method is known to show high sensitivity to the localization accuracy, it is necessary to check which characteristics the ML-3D NDT scan-matching has for changes in these initial conditions (initial pose, environmental layout) compared with the original 3D NDT scan-matching. Therefore, the scenario consists of: (1-1, 2-1) set the initial pose of MR to the ground-truth state confirmed by Isaac Sim, (2-2) apply a distance error from 20 cm to 60 to the ground-truth state. Scenarios 1 and 2 start differently from the scenes shown in Figure 5a,b below but proceed the same from Figure 5c–i. It should be noted that Isaac Sim published an accurate pose in the map through the ‘ROS1 Publish Transform Tree’, where the pose refers to the pose of the mobile prim for the world prim, as shown in Figure 5j. Table 1 below shows the internal parameters of ML-3D NDT scan-matching in this study and Table 2 shows the result of the comparative study in terms of localization accuracy and layer-by-layer scan-matching score. Figure 6 presents the result of the comparative study in terms of localization accuracy and layer-by-layer scan-matching score.

Figure 6 shows the results of localization through 3D NDT and ML-3D NDT scan-matching for each scenario, scenarios 1-1 and 2-1. It can be seen that the 3D NDT scan-matching score, which is a smaller-the-better index, in the 1st layer changes whenever an object-teleport event occurs within the 1st layer area. It can be seen that the score of the 1st layer is the highest, and when it returns to its original scene, as shown in Figure 5i, it can be confirmed that the scores of all layers return to a similar level. If the score is lowered according to the event, the surrounding environment has changed to be more similar to the original scene by the corresponding teleport event. For example, while changing from Figure 5g to Figure 5h, the scores of other layers do not change; only the score of the 1st layer decreases. This result shows that rejecting a specific layer with a high probability of environmental change in a logistics warehouse environment from 3D NDT scan-matching is feasible and significant. According to the results of Figure 6 and Algorithm 1, it was confirmed that the mean distance error of ML 3D NDT scan-matching was reduced by about 21% compared to the mean of 3D NDT, and the mean of heading error was also reduced by about 40%. There was no significant difference by scenario.

**Figure 7:**
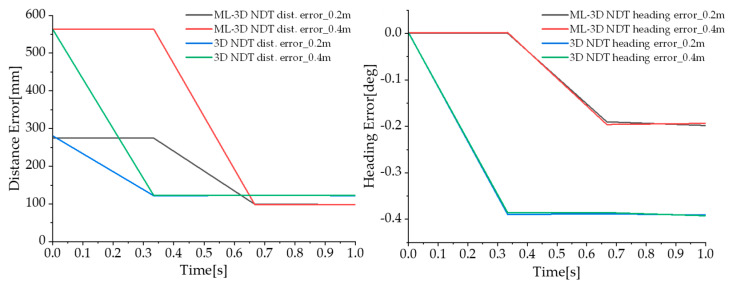
Result of the scenario 2-2 in terms of localization accuracy.

In our study, we evaluated the performance of our proposed method in the presence of distance errors in the initial pose, demonstrating its ability to perform accurately and consistently under different initial pose conditions. Figure 7 and Table 3 show the results for scenario 2-2. Even when distance errors of 20 cm, 40 cm, and 60 cm were applied to the initial state, it was confirmed that the distance error and heading error of the ML 3D NDT were approximately 18% and 47% lower than that of the 3D NDT, respectively. This result confirmed that the ML 3D NDT method is excellent in robustness against uncertainty in the initial state. Our findings also suggest that the proposed method can be a highly effective and reliable solution for warehouse localization tasks, even in significant initial state errors.

## 4. Conclusions

This paper proposed a multi-layered 3D NDT scan-matching approach for robust localization even in the highly dynamic environment of warehouse logistics. It is essential to note the explicit assumptions of this study, namely that a 3D point cloud map and initial pose are required before starting the scan-matching process. While this study did not specifically address the challenges of repetitive or uniform structures in warehouses, the proposed method can contribute to the overall localization framework by reducing uncertainties in scan-matching. In this approach, we partitioned a given 3D point-cloud map and the 3D lidar scan measurements into several layers to perform the 3D NDT scan-matching by separating the static and dynamic zones since slicing the 3D point-cloud map in the height direction to separate significant environmental changes can be effective if the environmental changes are mainly confined to a specific height range near the ground as in the warehouse. In order to confirm the significance of the proposed method, Omniverse Isaac Sim-integrated simulation was conducted for four scenarios in this study. Compared with 3D NDT scan-matching, the proposed method showed a mean distance error reduction of around 20% and a mean heading error reduction of approximately 48.7%, even in the computational complexity is increased by 10.9% and logistics warehouse showing significant environment changes from a number of the object-teleport events. In our future work, we aim to investigate the impact of different warehouse layouts, logistics products and storage policy [27,28] on the performance of the proposed multi-layered 3D NDT scan-matching method. To achieve this, we plan to conduct case studies to provide practical insights into the capabilities and limitations of our method and identify the appropriate layout and type of goods for a warehouse equipped with our method. By doing so, we believe we can better understand the potential benefits and challenges of implementing our method in real-world warehouse environments. Additionally, we plan to improve our method’s computational cost and localization accuracy by studying how to adaptively determine the layer’s height to environmental changes based on scan-matching scores and object classification methods. We also plan to explore the application of our method to larger and more complex warehouse environments and investigate the integration of our method with other localization techniques for increased accuracy and reliability. Furthermore, we aim to develop a real-time implementation of our method to enable its use in industrial settings. Overall, we believe that our proposed method has the potential to improve the efficiency and safety of warehouse operations significantly. We hope our work will inspire further research in this field and contribute to developing more advanced and effective warehouse localization methods. 

## Figures and Tables

**Table 1:** Internal parameters of ML-3D NDT scan-matching with three layers.

Matching Resolution ^1^	Down Sampling Rate [%]	Voxel Size ^2^ [m^3^]	Transform Epsilon	Max. Iteration	Distance Metric ^3^
1.0	50	0.5 × 0.5 × 0.5	0.1	35	Euclidean distance

^1^ The resolution of the voxel grid used for scan-matching. ^2^ The size of the voxels used for discretizing the point cloud. ^3^ The distance metric used for measuring the similarity between the scans and map.

**Table 2:** Result of the comparative study of 3D NDT and ML 3D NDT in scenario 1-1 and 2-1.

Method	Distance Error Mean [mm]	Distance Error Mean [mm]	Heading Error Mean [deg]	Heading Error Mean [deg]	Computational Complexity [ms]
	Scenario 1-1	Scenario 2-1	Scenario 1-1	Scenario 2-1	
3D NDT	124.3	124.3	−0.39	−0.39	202
ML 3D NDT	98.22 (−21.0%)	98.2 (−20.9%)	−0.24 (38.5%)	−0.23 (41.0%)	224 (10.9%)

**Table 3:** Result of the comparative study of 3D NDT and ML 3D NDT in scenario 1-2 and 2-2.

	20 cm		40 cm		60 cm
	ML-3D NDT	3D NDT	ML-3D NDT	3D NDT	
Distance error	101.7 (−18.6%)	125.0	103.2 (−18.3%)	126.3	divergence
Heading error	−0.205 (−47.4%)	−0.39	−0.21 (−46.1%)	−0.39	divergence

## Data Availability

The data used to support the findings of this study are available from the corresponding author upon request.
